# Same Same but Different: A Clinical Characterization of Men with Hypersexual Disorder in the Sex@Brain Study

**DOI:** 10.3390/jcm8020157

**Published:** 2019-01-30

**Authors:** Jannis Engel, Maria Veit, Christopher Sinke, Ivo Heitland, Jonas Kneer, Thomas Hillemacher, Uwe Hartmann, Tillmann H.C. Kruger

**Affiliations:** 1 Department of Psychiatry, Social Psychiatry and Psychotherapy, Hannover Medical School, Carl-Neuberg-Str. 1, 30625 Hannover, Germany; veit.maria@mh-hannover.de (M.V.); sinke.christoph@mh-hannover.de (C.S.); heitland.ivo-aleksander@mh-hannover.de (I.H.); kneer.jonas@mh-hannover.de (J.K.); thomas.hillemacher@klinikum-nuernberg.de (T.H.); hartmann.uwe@mh-hannover.de (U.H.); krueger.tillmann@mh-hannover.de (T.H.C.K.); 2 Department for Psychiatry and Psychotherapy, Paracelsus University Hospital Nuremberg, Prof. Ernst-Nathan-Str. 1, 90419 Nürnberg, Germany

**Keywords:** hypersexuality, sexual addiction, sexual compulsivity, phenomenology, comorbidities

## Abstract

Problems arising from hypersexual behavior are often seen in clinical settings. We aimed to extend the knowledge about the clinical characteristics of individuals with hypersexual disorder (HD). A group of people who fulfilled the proposed diagnostic criteria for HD (men with HD, *n* = 50) was compared to a group of healthy controls (*n* = 40). We investigated differences in sociodemographic, neurodevelopmental, and family factors based on self-report questionnaires and clinical interviews. Men with HD reported elevated rates of sexual activity, paraphilias, consumption of child abusive images, and sexual coercive behavior compared to healthy controls. Moreover, rates of affective disorders, attachment difficulties, impulsivity, and dysfunctional emotion regulation strategies were higher in men with HD. Men with HD seem to have experienced various forms of adverse childhood experiences, but there were no further differences in sociodemographic, neurodevelopmental factors, and family factors. Regression analyses indicated that attachment-related avoidance and early onset of masturbation differentiated between men with HD and healthy controls. In conclusion, men with HD appear to have the same neurodevelopment, intelligence levels, sociodemographic background, and family factors compared to healthy controls, but they report different and adverse experiences in childhood, problematic sexual behavior, and psychological difficulties.

## 1. Introduction

Hypersexual disorder (HD) is characterized by intense, repetitive sexual fantasies, urges, and behaviors that lead to clinically significant psychological impairment [1,2,3]. Kafka [3] proposed that hypersexual disorder should be included as a category in the Diagnostic and Statistical Manual of Mental Disorders, 5th edition (DSM-5) [4], but the proposal was ultimately rejected. One of the reasons given was the lack of experimental research on hypersexual disorder [5,6]. In the forthcoming version of the International Classification of Diseases, ICD-11, hypersexual disorder will be classified as compulsive sexual behavior disorder [7].

Alarming numbers are shown by a recent representative study of men (*n* = 1151) and women (*n* = 1174) in the United States that found 10.3% of men and 7% of women showed clinically relevant levels of distress and/or impairment due to difficulties in controlling sexual urges, feelings, and behaviors [8]. Manifestations of hypersexual behavior can include both real-world sexual contacts and online sexual activities. Online use of sexual content in combination with masturbation is the most common behavior that leads to men being diagnosed with hypersexual disorder according to the Kafka criteria [3,9].

Cooper [10] pointed out that the triad of access, affordability, and anonymity enables people to access whatever content they like anonymously, regardless of economic and social constraints. Of course, internet usage patterns vary greatly between individuals with some engaging excessively in online sexual activities [11] whereas others use dating platforms to find partners for sexual encounters [12]. The main driving forces for excessive online sexual activity may be the anticipated and experienced gratification associated with sexual arousal and the accessibility of virtually all types of sexual stimulus [13]. 

Little is known about the clinical characteristics of people with HD. Data from a study without a control group suggest that most subjects with men with HD are in intimate relationships, educated, and employed [14]; however, many also report intimacy deficits due to disengagement from family and a history of sexual, physical, and/or emotional abuse [15]. Intensive use of pornography [16,17] and hypersexual behavior in general [18] have been linked to risky sexual behaviors. Studies indicate that psychiatric comorbidities, particularly mood disorders, are prevalent in HD with rates ranging from 72%–90% in the case of mood disorders [14,19,20,21], and 42% in the case of substance use disorders [22]. Findings on the relationship between hypersexual disorder and impulsivity are mixed. Two studies [23,24] of treatment-seeking individuals fulfilling the proposed criteria for hypersexual disorder [3] found that between 48% and 53.3% displayed elevated impulsivity in self-report measures. Reid, Berlin, and Kingston [25] suggested that a context-specific form of sexual impulsivity, but not general impulsivity, might be prevalent in hypersexual disorder. Hypersexual behavior has been shown to be associated with neuropsychological impairments and alterations in attentional bias [26] and executive control [27,28].

From a biological perspective, the testosterone system plays a crucial role for the development and maintenance of sexual behavior [29]. As a marker of prenatal androgen exposure, the ratio of the lengths of the second and fourth digits (2D:4D) can be used, and there is some evidence that a lowered 2D:4D ratio might be connected to hypersexual behavior [30], although mixed findings have been reported. Some studies of the general population have demonstrated that a lower 2D:4D ratio (a more masculine pattern) is linked to having a higher number of sexual partners and more offspring [30,31,32], whereas others have shown that a high 2D:4D ratio is linked to promiscuity in men [33].

The aim of this study was to investigate the clinical and some specific (neuro-)developmental characteristics of men with hypersexual disorder in a large sample of people who fulfill the proposed diagnostic criteria [3] and compare them with healthy controls. Furthermore, detailed analyses should identify potential risk factors contributing to hypersexual behavior, such as biographical factors, i.e., adverse childhood events and attachment difficulties [34], as well as early age of sexual interest [35]. We present data on parameters not previously measured in comparable samples and we discuss the results in the light of the current understanding of hypersexuality.

## 2. Experimental Section

### 2.1. Recruitment

#### 2.1.1. Hypersexual Disorder Group

**Figure 1:**
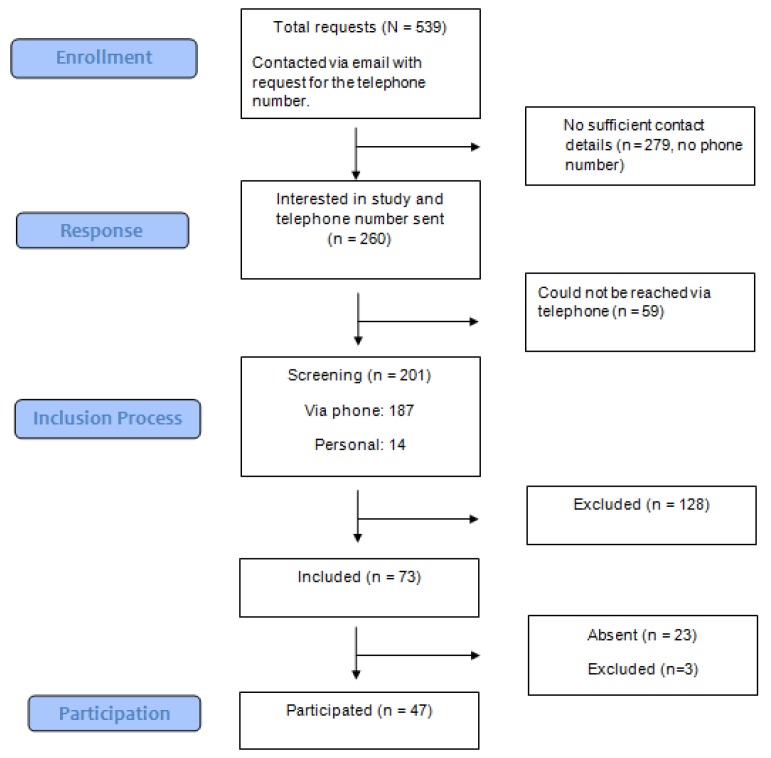
Recruitment of the hypersexual disorder group.

Men with HD were recruited between December 2016 and August 2017 through a press release by the Section of Clinical Psychology and Sexual Medicine, Department of Psychiatry, Social Psychiatry, and Psychotherapy at Hannover Medical School, Germany. The press release was taken up by local newspapers and social media (e.g., www.facebook.com, www.instagram.com) and resulted in 539 self-identified men with HD expressing an interest in participating in the study (see Figure 1). Two-hundred-and-sixty men responded to an email asking for a telephone number. Fifty-nine of the 260 individuals who provided a telephone number could not be reached by telephone, but the remaining 201 were screened for hypersexual disorder in a semi-standardized telephone interview of about 45 minutes carried out by a trained psychologist using Kafka’s [3] proposed criteria. Individuals were eligible for the study if they fulfilled Kafka’s [3] proposed criteria for hypersexual disorder. The questionnaires used in this study were sent by mail to eligible participants. Three participants whose scores did not reach the cut-off (53) of Hypersexual Behavior Inventory 19 [36] were excluded post hoc. Kafka’s [3] criteria for hypersexual disorder consist of clinically significant symptoms that arise from sexual urges, fantasies or behaviors, and recur over a period of 6 months that individuals struggle to control and are not due to the direct physiological effect of an exogenous substance. Seventy-three of the 201 individuals who were screened met these criteria and were deemed eligible for the study; 50 decided to participate and they formed the hypersexual disorder group (HD group, see Figure 1 chart). 

#### 2.1.2. Healthy Controls 

**Figure 2:**
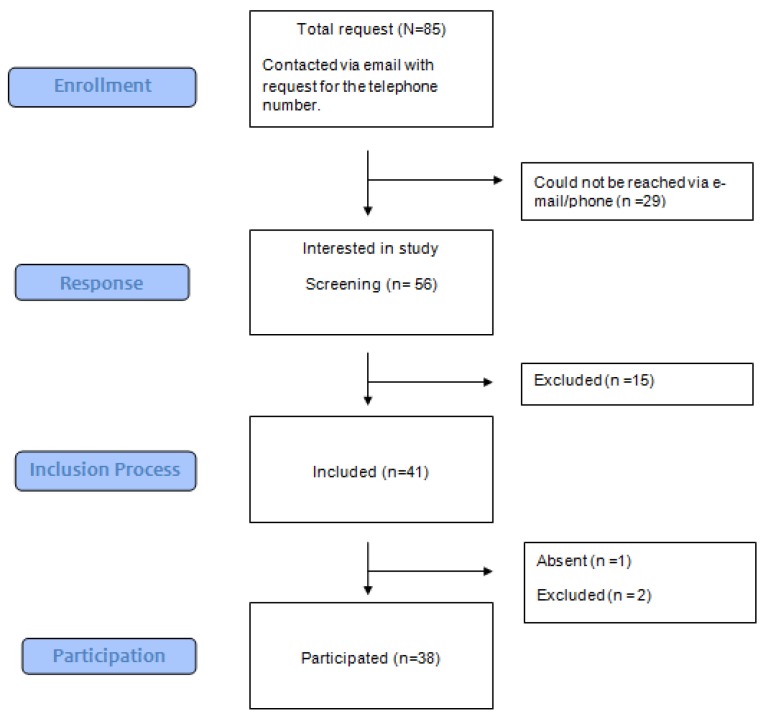
Recruitment of healthy controls.

Healthy controls were recruited via advertisements on the Hannover Medical School, Germany, intranet homepage. Eighty-five individuals responded to the advertisements (see Figure 2) of whom 56 responded to an email asking for a telephone number. Twenty-nine of these 56 could not be reached via telephone for screening. The controls were matched for age (*p* = 0.587) and education (*p* = 0.503) with HD group. Data from two healthy controls were subsequently excluded from analysis (one reported a severe head injury prior to study participation, one reported a homosexual orientation, and one control participant did not show up to assessment). 

#### 2.1.3. Exclusion Criteria

Exclusion criteria for all participants were: intellectual disability (as measured by Wechsler Adult Intelligent Scale-IV), a psychotic disorder (assessed with Structured Clinical Interview for DSM-IV Axis 1 disorders, SCID-I), severe head injury, homosexual orientation on the Kinsey scale, and pedophilic sexual preference (assessed in a semi-structured interview). In our Sex@brain project we focused on heterosexual participants due to the heterosexual nature of the stimuli in the upcoming experiments. All participants declared that their primary sexual interest was in women although some reported a history of same-sex sexual contact. 

All participants provided written, informed consent before participating and received monetary compensation for participation. They were informed that they could withdraw from the study at any time. The study was conducted in accordance with the Declaration of Helsinki and was approved by the ethics commission of the Hannover Medical School, Germany. The results reported here were obtained as part of a larger assessment that included a neuropsychological test battery and functional magnetic resonance imaging.

### 2.2. Measures

The variables were classified into three categories: (1) sociodemographic, neurodevelopmental, and family factors, (2) sexual characteristics, and (3) psychological characteristics including psychiatric comorbidities. For an exact description of items please see the notes to Table 1, Table 2, Table 3 and Table 4.

#### 2.2.1. Sociodemographic, Neurodevelopmental, and Family Factors

A questionnaire was used to collect sociodemographic data, namely age, highest educational qualification, employment status, lifetime criminal history, and relationship status. There were also questions about neurodevelopmental perturbations, sibling position, parental health at birth, and maternal and paternal age at birth. Aversive childhood experiences were assessed with the Childhood Trauma Questionnaire (CTQ) [37]. The developmental and neurodevelopmental perturbations investigated were birth complications, prolonged bedwetting, delayed walking, delayed speech development, and childhood accidents leading to unconsciousness. Handedness was determined using a 10-item adaptation of the Edinburgh Handedness Inventory [38] and 2D:4D ratio was estimated using images obtained from a portable scanner. The lengths of digits of the right hand were estimated independently by two research assistants (inter-rater reliability: *r* = 0.83) and calculations were based on the means of the two ratings.

Intelligence was estimated from the four subtests of the fourth edition of the Wechsler Adult Intelligent Scale (WAIS-IV) [39] that are most highly correlated with full scale IQ as measured by the German WAIS-IV. These four subtests are Vocabulary (verbal comprehension; *r* = 0.7), Block Design (perceptual reasoning; *r* = 0.65), Arithmetic (working memory; *r* = 0.73), and Coding (processing speed; *r* = 0.5). 

#### 2.2.2. Sexual Characteristics

Sexual development and behavior were assessed via a semi-structured interview and a set of questionnaires. We collected data on age at first ejaculation, masturbation in the week prior to assessment (duration and frequency), intercourse in the week prior to assessment, and lifetime total of sexual partners. Moreover, we assessed duration and frequency of pornography consumption, number of affairs, paraphilias, sexual coercive behavior, consumption of child abuse images, and sexual dysfunctions. Specific instruments were used to measure sexual excitation and inhibition proneness (Sexual Excitation Scale, SES and Sexual Inhibition Scale, SIS) [40], symptoms of hypersexual disorder (Hypersexual Behavior Inventory-19, HBI-19) [36], symptoms of cybersex addiction (Internet Addiction Test for online sexual activities—short version, sIATsex; [41] and sexual addiction (Sexual Addiction Screening Test-Revised, SAST-R) [42]. 

#### 2.2.3. Psychological Characteristics and Comorbidities 

Psychiatric comorbidities were diagnosed using the German version of the SCID-I [43]. Additional questionnaires were used to assess impulsivity (Barrat Impulsiveness Scale-11, BIS-11) [44], substance abuse (Fagerström Test for Nicotine Dependence, FTND) [45], hazardous and harmful patterns of alcohol consumption (The Alcohol Use Disorder Identification Test, AUDIT) [46], depressive symptoms (Beck Depression Inventory-II, BDI-II) [47], bonding (Experiences in Close Relationships-Revised, ECR-R) [48], alexithymia (Toronto Alexithymia Scale, TAS-26) [49], and emotion regulation (ERQ, Emotion Regulation Questionnaire [50]; Fragebogen zur Erhebung der Emotionsregulation, FEEL-E [51]. 

Attention deficit hyperactivity disorder (ADHD) was diagnosed on the basis of scores ≥15 on both the Wender Utah Rating Scale (WURS-K) [52] and ADHD self-assessment scale (ADHS-SB) [53]. 

#### 2.2.4. Logistic Regression Analysis

To identify possible predictive factors for hypersexual disorder we carried out a binary logistic regression analysis with group classification as dichotomous dependent variables. Our aim was to identify factors that differentiated between men with HD and healthy controls. The number of independent variables was chosen on recommendations by Agresti [54] (p. 138). 

### 2.3. Data Analysis

All analyses were executed with SPSS Statistics Version 24 (IBM^®^ Corporation, Amonk, NY, USA). Analyses were carried out using independent *t*-tests, Mann–Whitney *U* tests or Fisher’s exact tests for dichotomous variables. Fisher tests for tables larger than 2 × 2 were also used, as all polytomous categorical variables had at least one expected cell frequency of less than 5. As this was one of the first extensive phenomenological studies that included both men with hypersexual disorder and healthy controls in the search for group differences regarding the theoretically derived set of clinical variables tested here, we opted for an exploratory approach and report two-tailed significance levels without correction for multiple comparisons (all analyses *p* < 0.05). However, for interested readers we also included Bonferroni corrected significance in Table 1, Table 2, Table 3 and Table 4. Effect sizes for parametric tests were expressed as Cohen’s *d*, with *d* = 0.2 indicating a small effect, *d* = 0.5 a medium effect, and *d* = 0.8 a large effect [55]. There are variations in group sizes on the various tests because questionnaires with missing data were excluded from analysis. To control for the effects of psychiatric disorders other than hypersexual disorder, all group comparisons were also computed after excluding participants with a history of any SCID-I diagnosis; this procedure yielded an *N* of 45 (HD = 21; HC = 22). The results of these analyses are presented in the Supplementary Materials.

## 3. Results

### 3.1. Sociodemographic, Neurodevelopmental, and Family Factors

As intended by subject matching there were no group differences in the sociodemographic variables regarding age (*t*(83) = 0.55, *p* = 0.587) and highest educational qualification (Fisher’s exact test (*N* = 85), *p* = 0.503; see Table 1). Also, employment status (Fisher’s exact test (*N* = 85), *p* = 0.458), lifetime criminal history (Fisher’s exact test (*N* = 85), *p* = 0.368), and relationship status (Fisher’s exact test (*N* = 85), *p* = 0.128) were not different between groups. There were also no differences in scores on the four WAIS-IV subscales used including the subtests vocabulary (*t*(82) = −1.28, *p* = 0.204), block design (*t*(82) = 0.92, *p* = 0.359), arithmetic (*t*(82) = 0.112, *p* = 0.911), and coding (*t*(82) = 1.66, *p* = 0.100), indicating similar intelligence levels among groups.

Indicators of neurodevelopmental perturbations were similar in men with HD and healthy controls including general developmental factors during childhood (Fisher’s exact test (*N* = 82), *p* = 1) distribution of handedness (Fisher’s exact test (*N* = 85), *p* = 0.645) and 2D:4D finger length ratio (*t*(77) = 0.34, *p* = 0.738). 

Our data show that men with HD and healthy controls grew up in families with similar structural family factors such as number of children in the household in which the participant grew up (*t*(78) = 0.01, *p* = 0.995); position in the birth order (*w*(78) = 718, *z* = −0.402, *p* = 0.687); position among children in the household (*w*(78) = 750, *z* = −0.464, *p* = 0.642); maternal age at birth (*t*(79) = 0.88, *p* = 0.384); and paternal age at birth (*t*(73) = 0.09, *p* = 0.93). Men with HD reported more frequently maternal psychiatric problems (Fisher’s exact test (*N* = 62), *p* = 0.001), but not paternal psychiatric problems (Fisher’s exact test (*N* = 68), *p* = 0.307) than healthy controls. Furthermore, the aversive childhood memories of men with HD differed substantially from healthy controls. Men with HD reported elevated rates of overall adverse childhood experiences (CTQ; *t*(68) = 2.71, *p* = 0.009, *d* = 0.57), in particular emotional abuse (*t*(73) = 3.53, *p* < 0.001, *d* = 0.73), emotional neglect (*t*(81) = 2.46, *p* = 0.016, *d* = 0.54), and sexual abuse (*t*(45) = 2.49, *p* = 0.017, *d* = 0.49) compared to healthy controls. However, physical abuse (*t*(80) = 1.60, *p* = 0.113) and physical neglect (*t*(83) = 1.49, *p* = 0.141) did not reach statistical significance. 

### 3.2. Sexual Characteristics

The sexual history from men with HD differed substantially from healthy controls (see Table 2). First of all, men with HD had earlier sexual experiences than control group. Men with HD reported that they were over a year younger when they started masturbating (*t*(79) = 3.59, *p* < 0.001, *d* = 0.80) and about a year younger when they first ejaculated (*t*(77) = 2.79, *p* = 0.007, *d* = 0.63). But they did not differ in age of first intercourse (*t*(83) = 1.868, *p* = 0.065). Men with HD and healthy controls reported similar duration of last/current relationship in months (*t*(42) = 0.14, *p* = 0.886), and number of children (*w*(75) = 728, *z* = −0.081, *p* = 0.936). However, men with HD differed in their sexual relationships from healthy controls. On average men with HD reported about eighty more female sexual partners (*w*(79) = 470.5, *p* = 0.001) and female coital partners (*w*(81) = 443, *p* < 0.000) than healthy controls. Moreover, despite their predominant heterosexual orientation, men with HD reported sexual activities with men with more male sexual partners (*w*(83) = 567.5, *p* < 0.000) and male coital partners (*w*(83) = 664, *p* = 0.002), whereas healthy controls reported almost no sexual activities with men. Moreover, men with HD were more likely to report that they had an affair during their last or current relationship (Fisher’s exact test (*N* = 81), *p* < 0.001), with 67% reporting an affair compared to only 19% in healthy controls. Furthermore, men with HD report more received problems through online sexual activities than healthy controls indicated by a group difference in sIATsex score (*t*(80) = −11.70, *p* < 0.001, *d* = 2.45). Accordingly, they reported that they consumed pornography more often in the week before the assessment (Fisher’s exact test (*N* = 84), *p* < 0.001), about 85% of men with HD reported at least three times of pornography consumption per week, compared to about 40% in healthy controls. Moreover, men with HD watched on average about seventy minutes more of pornography (*t*(47) = −3.61, *p* = 0.001, *d* = 0.73) than healthy controls. Duration of pornography consumption varied greatly between groups, with more than half of men with HD watching over an hour per week, compared to only 9% in healthy controls. Relating to sexual excitation and inhibition, men with HD reported more pronounced sexual excitation (SES: *t*(83) = 5.01, *p* < 0.001, *d* = 1.09), a lower sexual inhibition due to threat of performance consequences (SIS2: *t*(83) = −3.75, *p* < 0.001, *d* = 0.82). However, men with HD showed a higher score for perceived threat of performance failure (SIS1; *t*(80) = 2.30, *p* = 0.024, *d* = 0.48). Interestingly, the prevalence of reported sexual dysfunction was similar in men with HD and healthy controls (Fisher’s exact test (*N* = 85), *p* = 0.765), specifically there were no differences in erectile disorder, hypoactive desire disorder, premature and delayed ejaculation. 

Paraphilias like exhibitionism, voyeurism, masochism, sadism, fetishism, frotteurism or transvestism were more prevalent in men with HD (Fisher’s exact test (*N* = 85), *p* < 0.001) (see Table 3). Men with HD were also more likely to report sexually coercive behavior (Fisher’s exact test (*N* = 85), *p* < 0.001) and a higher rate of having consumed images of child abuse at least once in their lives (Fisher’s exact test (*N* = 82), *p* = 0.009); none of the healthy controls reported having consumed child abuse images.

### 3.3. Psychological Characteristics and Comorbidities

Most importantly, men with HD revealed more often psychiatric symptoms such as depression, impulsivity or symptoms of ADHD (see Table 4). Separate analysis of current diagnoses of SCID-I subcategories revealed a higher rate of affective disorders in the HD group (Fisher’s exact test (*N* = 85), *p* = 0.015). This increased rate of diagnoses was supported by the psychometric assessment of depressive symptoms with higher symptoms in men with HD (BDI-II; *t*(79) = 5.47, *p* < 0.001, *d* = 1.13). Rates of current SCID-I diagnosis of substance abuse and/or dependency were similar in the two groups (Fisher’s exact test (*N* = 85), *p* = 1.000), just as psychometric assessment of alcohol consumption (AUDIT; *t*(82) = −0.93, *p* = 0.354) and nicotine abuse (FTND; *t*(83) = 0.73, *p* = 0.471, *d* = 0.16). However, rates of current anxiety disorders (Fisher’s exact test (*N* = 85), *p* = 0.690), obsessive-compulsive disorders (Fisher’s exact test (*N* = 85), *p* = 1.000), and somatic symptoms and eating disorders (Fisher’s exact test (*N* = 85), *p* = 1.000) did not differ between the groups. Taken together, men with HD and healthy controls showed similar proportions of current SCID-I (Fisher’s exact test (*N* = 80), *p* = 0.104) and lifetime SCID-I diagnosis (Fisher’s exact test (*N* = 85), *p* = 0.190). However, men with HD were more likely to display symptoms of ADHD at the time of assessment (ADHS/SB; *t*(73) = 6.31, *p* < 0.001, *d* = 1.37) and to report childhood symptoms of ADHD (WURS-K; *t*(82) = 3.76, *p* < 0.001, *d* = 0.82), Moreover, men with HD revealed greater impulsivity than healthy controls (BIS-11; *t*(81) = 3.76, *p* < 0.001, *d* = 0.83). The results relating to emotion regulation were mixed: men with HD were more likely to use maladaptive emotion regulation strategies (FEEL-E-maladaptive strategies; *t*(81)= 3.54, *p* < 0.001, *d* = 0.78) and “reappraisal” strategies (ERQ: Reappraisal; *t*(83) = −2.477, *p* =.015, *d* = 0.545) but use of adaptive strategies (FEEL-E-adaptive strategies; *t*(81) = −1.26, *p* = 0.212) was similar as was use of the “suppression” strategies (ERQ: Suppression; *t*(83) = 1.852, p = 0.068). Men with HD reported more symptoms of alexithymia (TAS-26; *t*(79) = 4.11, *p* < 0.001, *d* = 0.92) elevated scores in both, attachment-related anxiety (ECR-R anxiety: *t*(78) = 5.413, *p* < 0.000, *d* = 1.245) and attachment-related avoidance (ECR-R avoidance: *t*(82) = 4.908, *p* < 0.000, *d* = 1.064). 

### 3.4. Logistic Regression Analysis 

The variables that differentiated best between men with HD and healthy controls were age at onset of masturbation (OR = 0.55, 95% CI (0.35, 0.86)) and avoidant attachment style (*OR* = 1.06, 95% CI (1.01,1.11)). Non-significant were child traumata and anxious attachment style. The specified regression model had a good fit (with Nagelkerke *R*^2^ = 0.55 and Hosmer–Lemeshow Test: *χ*^2^(7) = 11.76, *df* = 7, *p* = 0.11) and explained about 55% of the variance between the two groups. The mean classification accuracy was 80.0% (78.1% specificity, 81.4% sensitivity).

## 4. Discussion

This study is one of the first to analyze phenomenological data from a large sample of individuals who met the proposed criteria for hypersexual disorder [3] and compare them with a group of healthy controls. A considerable number of sociodemographic, neurodevelopmental, and family factors, as well as sexual characteristics, psychological characteristics, and comorbidities were investigated. 

Through analysis of an extensive set of variables this study has revealed important differences between people diagnosed with hypersexual disorder and healthy controls. 

In summary, men with HD seem to have experienced more difficulties during childhood than healthy controls, being more likely to have had a mother with psychiatric problems, to have experienced various forms of adverse experiences during childhood and to have displayed symptoms of childhood ADHD. Moreover, attachment difficulties with pronounced avoidance in close relationships were higher in men with HD. Onset of masturbation was at an earlier age in men with HD and they experienced higher sexual excitation and less sexual inhibition due to concern about negative consequences, but higher sexual inhibition due to threat of performance failure. Furthermore, men with HD were characterized by problems arising through subjective complaints through their high use of online sexual activities and reported more deviant sexual behaviors, namely higher rates of paraphilia, sexually coercive behavior, and consumption of images of child abuse. Diagnoses of affective disorders and symptoms of a large set of psychiatric comorbidities such as impulsivity, symptoms of adult ADHD, alexithymia, and maladaptive emotion regulation strategies were increased in men with HD. 

There were indicators of differences in the childhood of men with HD compared to healthy controls. In our sample, dysfunctional emotion regulation strategies such as a lowered reappraisal and increased maladaptive strategies can be seen in men with HD, as well as increased alexithymia. Men with HD reported a higher rate of adverse childhood experiences; especially the rates of emotional abuse and neglect, as well as sexual abuse were increased, which have been shown to be associated to emotion regulation difficulties [57]. Moreover, maladaptive emotion regulation strategies in men with HD may be fostered by the psychiatric difficulties experienced by the child’s mother [58] which were increased in men with HD. We argue that a possible path to HD is via a series of aversive states and experiences in childhood and adolescence which facilitates the development of maladaptive emotion regulation strategies [34]. Moreover, dysfunctional emotion regulation strategies may be associated to the attachment difficulties we observed in men with HD, as children show dysfunctional emotion regulation strategies when they are in a non-secure attachment to their mothers [59]. In a representative survey of the German population, use of online sexual activities was significantly associated to anxiously attached individuals [60]. Our regression analysis showed that avoidance in close relationships differentiated between men with HD and healthy controls, which is in line with Katehakis’s [34] suggestion that some HD patients may have disengaged emotionally during childhood. This may lead to impaired development of the limbic system and parts of the prefrontal cortex, due to an adverse interaction involving the central nervous system, autonomic central nervous system, and hypothalamic–pituitary–adrenal axis [34].

Our findings are in line with findings suggesting that men with HD experience deficits in affect regulation and negative affect and may use hypersexual behavior as a maladaptive coping strategy [61]. These neurobiological deficits may develop in early childhood and may impair emotional and intellectual abilities [34]. However, we found only emotional disabilities and no differences in intelligence as measured by WAIS-IV subtests [39] were observed in this study and in a study with a smaller sample [62]. 

A disposition to hypersexual behavior may manifest early in sexual development, our HD group was characterized by an early onset of masturbation which differentiated significantly between men with HD and healthy controls in logistic regression analysis. Moreover, hypersexual behavior has been associated to early onset of sexual interest [35], and early onset of sexual behavior has been linked to sensation-seeking behavior, depression, and anxiety [63]. Frequency and duration of pornography consumption were higher in men with HD. However, it is important to note that not only the quantity of pornography consumption results in problems but that the relationship between frequency and duration of pornography use and treatment-seeking is not linear, but mediated by the severity of perceived negative symptoms associated with use of pornography [64]. The incentive salience theory of addiction [65,66], which has been applied to HD [26,62], posits that in addiction “wanting” stimuli becomes dissociated from “liking” stimuli. This could explain why men with HD continue with problematic behavior despite the perceived negative consequences. In fact, the men with HD in our sample report more problems due to their increased pornography consumption.

The important role of sexual excitation and inhibition in hypersexual behavior has been shown in large surveys [35,67]. The HD group in our sample reported higher sexual excitation and less sexual inhibition due to perceived threat of performance consequences, and thus higher sexual arousal. We argue that this specific pattern of sexual arousal is a vulnerability factor which, in combination with using sexual behavior as a dysfunctional emotion regulation strategy, increases the likelihood of developing hypersexual disorder. A study of a large online sample that used total number of sexual outlets as an indicator of sex drive found that high sexual interest was associated with self-reported consumption of images of child abuse [68]. In fact, in our sample no healthy control reported to have ever consumed child pornography as opposed to 80% of men with HD. Rates of sexual coercive behavior were increased in men with HD, showing highly increased rates of consumption of child abusive images in men with HD. Based on these results combined with meta-analyses that found hypersexuality to be an empirically supported risk factor in sexual recidivism [69], we encourage clinicians to assess criminal history and potential sexual coercive behavior in patients with HD. 

Furthermore, we found increased rates of paraphilic interest in men with HD. To date, there are inconsistent findings on the association of paraphilic interests and HD. Some studies suggest increased rates of paraphilic interests [14], whereas in a field trial for the proposed criteria of HD [9] no connection was found. A possible explanation for divergent rates would be openness to report paraphilic interests, because in Germany information and data gathered in the course of research and treatment situations are protected by confidentiality, even when they include reports on paraphilic interest, child pornography consumption, and sexual coercive behavior. Paraphilic interest by itself (if no others are harmed) does not require or justify clinical intervention [4]; however, paraphilic interests are often associated with relationship difficulties [70]. Generally, the psychological burden represented by HD is one of the main findings to emerge from this study. Our data underline increased symptoms of some psychiatric comorbidities in HD. Especially, the diagnoses of both current and lifetime symptoms of affective disorders are increased in HD group. In our study, the score for symptoms of depression as measured by BDI-II was almost three times as high in men with HD as in healthy controls. In line with our findings, Weiss [71] found that the prevalence of depression was almost 2.5 times higher in men with HD than in the general population. Together the results of a range of studies investigating comorbid affective disorders in hypersexual disorder suggest the prevalence is between 28% and 42% [20,70,71]. Moreover, we suspect that impulsivity, particularly context-specific sexual impulsivity [25] is a characteristic of hypersexual disorder, based on our observation of increased impulsivity in men with HD and future studies should attempt to investigate this. Substance abuse is often connected to increased impulsivity. In our sample we found only increased impulsivity with a large effect size, but the rates of substance abuse did not differ between groups. There are theoretical and empirical studies suggesting that substance abuse plays a role in hypersexual behavior [22,72,73], but the picture remains unclear, since different studies have used different measures and sample sizes. Furthermore, future studies should investigate potential risky sexual behaviors in men with HD, which have been shown to be associated to a large variety of mental disorders [74].

**Figure 3:**
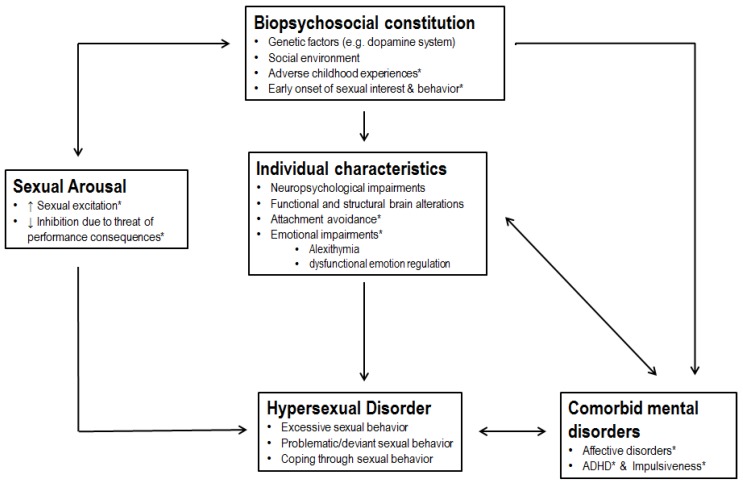
Working model of hypersexual disorder. We assume an underlying combination of genetic and environmental factors that may increase the likelihood of developing hypersexual disorder. A combination of biopsychosocial factors, e.g., genetic and epigenetic factors and adverse childhood events shape individual characteristics and increase the likelihood of developing comorbid psychiatric disorders. A high sexual arousal may be connected to genetic factors and may be both influenced by and influence early onset of sexual experiences. Dysfunctional characteristics of the individual, comorbid disorders, and high sexual arousal may lead to the development of hypersexual disorder. The factors marked with an asterisk were derived a posteriori from our results.

Based on theoretical assumptions and our results, we created a working model for the etiology of hypersexual behavior (Figure 3). While there is no evidence of a monocausal etiology of hypersexual disorder, the model points out multiple components that may increase the possibility of developing hypersexual disorder. This working model may be useful for generating new research questions and adaptions of treatment programs. 

Our data have several implications for treatment. We suggest that clinicians assess possible emotional abuse and neglect, as well as sexual abuse in men with HD. Moreover, our data show that symptoms of comorbid adult ADHD were increased in men with HD and it has been suggested that these patients are likely to benefit from pharmacotherapy and behavioral therapy combined [75]. As a reduction of the use of dysfunctional emotion regulation strategies was seen in our sample, a cognitive-behavioral therapy should also focus on dysphoric mood states and impulsivity in men with HD [76]. A non-judgmental therapeutic approach is needed to tackle paraphilia, which is more frequent in men with HD. We found increased rates of sexual coercive behavior and consumption of child abusive images in men with HD, and if not restricted by limits of confidentiality, we suggest that an assessment by clinicians is strongly advised to prevent possible harmful behavior. 

## 5. Limitation

It is important to note that that this sample consisted of individuals who volunteered to take part in a clinical study and agreed to report intimate details of life events, inner experiences, and sexual behavior. Thus, the characteristics of this sample may not be comparable to those of people with hypersexual disorder who are reluctant to share private information.

Causal explanations about the etiology of HD are difficult to draw, because—with the exception of 2D:4D ratio—we relied on self-report data and clinical interviews in a cross-sectional study and responses may have been affected by social desirability bias.

It is difficult to transfer the conclusions of this study to other cultures. Furthermore, this Western European sample was not representative of the Western European population in terms of, for example, age and educational level. 

## 6. Conclusions

Men with HD appear to have the same neurodevelopment, intelligence levels, sociodemographic background, and family factors compared to healthy controls. However, men with HD report differences in important areas of life, such as adverse experiences in childhood, problematic sexual behavior, and increased psychological difficulties.

## Figures and Tables

**Table 1:** Sociodemographic, neurodevelopmental, and family factors.

	Hypersexual Disorder Group (*n* = 47)		Healthy Volunteers (*n* = 38)		*p*-Value	*d*
**Sociodemographic Variables**	%	M (SD)	%	M (SD)		
Age		36.51 (11.47)		37.92 (12.33)	0.587 ^a^	
Highest educational qualification ^b^						
no school-leaving qualification	2		0		0.503 ^c^	
secondary school leaving certificate of secondary education 4 years	4		3			
secondary school leaving certificate (5 years)	11		5			
completed apprenticeship	28		26			
secondary school leaving certificate (8 years)	21		40			
university degree	34		26			
Employment status ^d^						
unemployed	9		14		0.458 ^c^	
in training	27		30			
retired	4		8			
employed	66		48			
Lifetime criminal history (yes) ^e^	19		11		0.368 ^c^	
Current intimate relationship (yes)	43		61		0.128 ^c^	
**Neurodevelopmental Factors**						
Developmental perturbations (yes) ^f^	43		42		1 ^c^	
Handedness ^g^						
right	83		79		0.645 ^c^	
left	4		10.5			
ambidextrous	13		10.5			
2D:4D finger length ratio ^h^		0.97 (.33)		0.96 (0.29)	0.738 ^a^	
**Family Factors**						
Number of siblings		1.51 (1.42)		1.51 (0.93)	0.995 ^a^	
Position in the birth order ^i^		1.67 (0.95)		1.72 (0.82)	0.687 ^j^	
Position among children in the household ^k^		1.57 (0.94)		1.72 (0.82)	0.642 ^j^	
Age at participants birth						
Paternal		30.02 (8.41)		29.88 (4.57)	0.930 ^a^	
Maternal		27.64 (7.77)		26.35 (4.79)	0.384 ^a^	
Childhood Trauma Questionnaire (CTQ) ^l,m^		57.42 (16.06)		49.97 (8.38)	0.009 ^a,^*	0.57
Emotional abuse		10.13 (4.76)		7.29 (2.51)	0.001 ^a,^†^,^*	0.73
Physical abuse		7.32 (3.67)		6.26 (2.38)	0.113 ^a^	
Sexual abuse		6.28 (2.38)		5.03 (3.42)	0.017 ^a,^*	0.49
Emotional neglect		11.74 (4.86)		9.24 (3.5)	0.016 ^a,^*	0.54
Physical neglect		7.34 (3.02)		6.53 (1.67)	0.141 ^a^	
Psychiatric problems ^n^						
Father (yes)	20		3		0.307 ^c^	
Mother (yes)	39		9		0.001 ^c,^†^,^*	
Intelligence [WAIS-IV] ^o^						
Vocabulary		46.26 (7.40)		43.97 (8.97)	0.204 ^a^	
Block-design-test		48.91 (9.68)		50.79 (8.77)	0.359 ^a^	
Arithmetic		17.15 (3.10)		17.24 (3.84)	0.911 ^a^	
Coding		66.67 (15.73)		71.92 (12.62)	0.1 ^a^	

Note. ^a^ Statistical analysis: *t*-test. ^b^ 0 = no school-leaving qualification; 1 = secondary school leaving certificate of secondary education (4 years); 2 = secondary school leaving certificate (5 years); 3 = completed apprenticeship 4 = secondary school leaving certificate (8 years); 5 = university degree. ^c^ Statistical analysis: Fishers exact test. ^d^ 0 = unemployed; 1 = in training; 2 = retired; 3 = employed. ^e^ Criminal status was assessed with a semi-structured interview (voluntary disclosure of confidential information) in which we asked participants to disclose all incidents of criminal behavior regardless of whether they had resulted in conviction. Lifetime history of any criminal behavior was coded 1; absence of criminal behavior was coded 0. ^f^ Assessed with a semi-structured questionnaire. Coded 1 if any of the following problems had occurred, otherwise coded 0: Complications at birth, problems with toilet training, problems with development of speech, problems with development of walking, head injuries, cranio-cerebral trauma, unconsciousness, childhood diseases (e.g., measles, mumps, rubella, diphtheria, chickenpox, poliomyelitis, meningitis, cerebral abscess, encephalitis and other illnesses resulting in a long stay in hospital). ^g^ Handedness was assessed using a 10-item adaptation of the German version of the Edinburgh Handedness Inventory [38]. ^h^ The participants’ right hands were photocopied to measure individual finger lengths. This was done by laying the surface of the palm of the right hand onto a photocopier, the photocopied image was then used to estimate the ratio. The basal crease where the finger joins the palm and the distal point of the fingertip were used as landmarks to assess length. 2D:4D ratio was calculated by two independent raters, by dividing the length of the second digit by the length of the fourth digit. The computed means of the two raters were used. ^i^ Position in birth order with regard to mother’s other children. (What position are you in the birth order of your full siblings and the half-siblings on your mother’s side?). ^j^ Statistical analysis: Wilcoxon–Mann–Whitney Test. ^k^ Position with regard to children growing up in the same household. (What position are you with regard to the siblings you grew up with?). ^l^ Five dimensions of childhood trauma were assessed via retrospective self-reports using the German version of the Childhood Trauma Questionnaire [37]. ^m^ Higher values indicate more problems. ^n^ Participants were asked about maternal and paternal psychiatric problems in a semi-structured interview. Presence was coded 1, absence 0. ^o^ Sum scores in the German version of the Wechsler Adult Intelligence Scale WAIS—Fourth Edition [39]. * *p*-values < 0.05 were considered significant. † significant after Bonferroni α-correction. In this section *p*-values < 0.002 (0.05/22) were considered as significant.

**Table 2:** Sexual characteristics.

	Hypersexual Disorder Group (*n* = 47)		Healthy Volunteers (*n* = 38)		*p*-Value	*d*
**Sexual History and Development**	%	M (SD)	%	M (SD)		
Onset of masturbation		11.16 (2.41)		12.97 (2.06)	<0.001 ^a,^†^,^*	0.8
Age at first ejaculation		11.91 (1.67)		12.81 (1.06)	0.007 ^a,^*	0.628
Age at first intercourse		16.57 (3.08)		17.71 (2.37)	0.065 ^a^	
Number of sexual partners ^b^						
Male		75.32 (376.12)		0.03 (0.16)	<0.001 ^c,^†^,^*	
Female		99.10 (211.10)		19.24 (22.00)	0.001 ^c,^†^,^*	
Number of coital partners ^d^						
Male		16.90 (76.52)		0.03 (0.16)	0.002 ^c,^*	
Female		86.71 (204.43)		15.00 (19.65)	<0.001 ^c,^†^,^*	
Number of relationships		4.81 (3.51)		5.35 (3.82)	0.506 ^a^	
Duration of last/current relationship [in month] ^e^		66.90 (99.24)		70.67 (73.48)	0.886 ^a^	
Number of children		0.74 (1.06)		0.77 (1.03)	0.936 ^c^	
Affairs in last/current relationship (yes)	67		19		<0.001 ^f,^†^,^*	
Consumption of pornography in the last week ^g^						
5	35.5		0		<0.001 ^f,^†^,^*	
4	17.8		10.8			
3	31.1		27			
2	6.7		21.6			
1	8.9		40.5			
Duration (minutes)		87.53 (125.50)		18.93 (19.82)	0.001 ^c,^†^,^*	0.73
Hypersexual Behavior Inventory (HBI-19) ^h,y^		72.37 (10.31)		30.26 (10.09)	<0.001 ^a,^†^,^*	4.123
Sexual Addiction Screening Test (SAST-R) ^I,y^		13.04 (3.20)		2.61 (2.71)	<0.001 ^a,^†^,^*	3.49
Short Internet Addiction Test—modified for cybersex (sIATsex) ^j,y^		39.62 (10.59)		17.11 (7.07)	<0.001 ^a,^†^,^*	2.45
Sexual excitation (SES) ^k,y^		60.92 (9.79)		50.41 (9.39)	<0.001 ^a,^†^,^*	1.093
Sexual inhibition ^k,y^ (SIS1/Threat of performance failure)		35.79 (8.18)		32.39 (5.39)	0.024 ^a,^*	0.481
Sexual inhibition ^k,y^ (SIS2/Threat of performance consequences)		25.66 (4.90)		29.45 (4.26)	<0.001 ^a,^†^,^*	0.819
Sexual dysfunctions (yes) ^l^	17		13		0.765 ^f^	

Note. ^a^ Statistical analysis: *t*-test. ^b^ Number of partners with whom the participant engaged in sexual behavior of any kind (including petting). ^c^ Statistical analysis: Wilcoxon–Mann–Whitney U Test. ^d^ Number of partners with whom the participant in vaginal or anal intercourse. ^e^ Participants were asked: How long has your current relationship lasted? or How long did your last relationship last? ^d^ Positive responses to the question Have you had/ did you have sex with others in your current/last relationship? were coded 1; negative responses were coded 0. ^f^ Statistical analysis: Fisher’s exact test. ^g^ Frequency of pornography consumption was classified as follows: 5 = Several times a day, 4 = once a day, 3 = several times a week, 2 = once a week, 1 = less than that. ^h^ Hypersexual behavior was assessed using the Hypersexual Behavior Inventory-19, for which the suggested cut-off score is 53 [36]. ^i^ Sexual addiction was assessed using the first 20 Items of the Sexual Addiction Screening Test-Revised [42]. ^j^ Cybersex addiction was assessed using an adaption of an Internet Addiction Test [41]. ^k^ Propensity to sexual excitation and inhibition was assessed using German versions of the Sexual Inhibition and Sexual Excitation Scales. Sexual inhibition was assessed using two independent subscales “Threat of performance consequences” and “Threat of performance failure” [40]. ^l^ All data given in this section based on the ICD-10 criteria for sexual dysfunctions. ^y^ Higher values indicate greater problems. * *p*-values < 0.05 were considered significant. † significant after Bonferroni α-correction. In this section *p*-values < 0.002 (0.05/22) were considered as significant.

**Table 3:** Sexual characteristics.

	Hypersexual Disorder Group (*n* = 47)		Healthy Volunteers (*n* = 38)		*p*-Value
	*n*	%	*n*	%	
Paraphilias (yes) ^a^	22	47	1	3	<0.001 †^,^*
Exhibitionism (yes)	3	6	0	0	0.25
Voyeurism (yes)	5	11	0	0	0.062
Masochism (yes)	7	15	0	0	0.015 *
Sadism (yes)	5	11	0	0	0.062
Fetishism (yes)	16	34	1	3	<0.001 †^,^*
Frotteurism (yes)	4	8	0	0	0.125
Transvestism (yes)	1	2	0	0	1
Sexual coercive behavior (yes) ^b^	33	70	8	21	<0.001 †^,^*
Lifetime consumption of images of child abuse (yes) ^c^	38	81	0	0	0.009 *

Note. Statistical Analysis: Fisher’s exact tests. ^a^ All data given in this section are based on the ICD-10 criteria for paraphilias. ^b^ Sexual violence was assessed using a four-item questionnaire asking about verbal assault, non-consensual sexual recordings, non-consensual touching/rubbing, and non-consensual penetration. ^c^ Consumption of images of child abuse was assessed with a semi-structured interview (voluntary disclosure of confidential information) in which participants were asked to disclose consumption regardless of whether this was known to the legal system. Any lifetime history of consumption of images of child abuse was coded 1; other responses were coded 0. * *p*-values < 0.05 were considered significant. † significant after Bonferroni α-correction. In this section *p*-values < 0.002 (0.05/22) were considered as significant.

**Table 4:** Psychological characteristics and comorbidities.

	Hypersexual Disorder Group (n = 47)		Healthy Volunteers (n = 38)		*p*-Value	d
	%	M (SD)	%	M (SD)		
**Lifetime Scid-i Diagnosis (yes) ^a^**	55		39		0.191	
**Current Scid-i Diagnosis (yes) ^a^**	43		25		0.104	
**Depression**						
Lifetime affective disorders (SCID-I) ^a^	50		13		<0.000 †^,^*	
Current affective disorders (SCID-I) ^a^	15		0		0.015 *	
Symptoms of depression (BDI-II) ^b,y^		17.25 (11.60)		5.92 (7.72)	<0.001 †^,^*	1.129
ADHD and impulsivity						
ADHD (ADHS-SB) ^c,y^		21.74 (11.26)		8.43 (7.45)	0.001 †^,^*	1.374
Childhood symptoms of ADHD (WURS-K) ^d,y^		26.59 (15.34)		15.00 (12.34)	0.001 †^,^*	0.824
Impulsivity levels (BIS-11) ^e,y^		68.24 (11.22)		59.62 (9.25)	<0.001 †^,^*	0.83
Substance abuse						
Lifetime substance abuse and/or dependency (SCID-I) ^a^	24		24		1	
Current substance abuse and/or dependency (SCID-I) ^a^	23		21		1	
Alcohol abuse (AUDIT) ^f,y^		6.57 (5.68)		7.68 (4.98)	0.354	
Nicotine dependence (FTND) ^g,y^		3.87 (6.23)		2.95 (5.3)	0.471	
Lifetime anxiety disorders (SCID-I) ^a^	11		8		0.724	
Current anxiety disorders (SCID-I) ^a^	8		5		0.687	
Lifetime obsessive compulsive disorder (SCID-I) ^a^	4		3		1	
Current obsessive compulsive disorder (SCID-I) ^a^	4		3		1	
Lifetime somatic problems, eating disorder or other (SCID-I) ^a^	4		0		0.499	
Current somatic problems, eating disorder or other (SCID-I) ^a^	2		0		1	
**Emotional Difficulties**						
Symptoms of alexithymia (TAS-26) ^h,y^		47.56 (10.31)		38.92 (8.35)	0.001 †^,^*	0.915
Maladaptive emotion regulation strategies (FEEL-E) ^i^		107.51 (21.3)		91.68 (18.97)	<0.001 †^,^*	0.781
Adaptive emotion regulation strategies (FEEL-E) i		115.42 (18.4)		120.77 (20.28)	0.212	
Emotion Regulation: Reappraisal (ERQ) ^j^		22.3 (8.62)		26.68 (7.45)	0.015 *	0.545
Emotion Regulation: Suppression (ERQ) ^j^		16.19 (5.16)		14.18 (4.72)	0.068	
Attachment style: Anxiety (ECR-R) ^k,y^		75.85 (22.84)		50.15 (18.17)	<0.000 †^,^*	1.245
Attachment style: Avoidance (ECR-R) ^k,y^		57.87 (13.7)		41.35(17.16)	<0.000 †^,^*	1.064

Note. Statistical analysis for SCID-I diagnosis: Fisher’s exact test, for all other analysis: *t*-test. ^a^ The Structured Clinical Interview for DSM-IV (SCID-I) [56] was used to determine the presence of psychiatric disorders. Presence of a disorder was coded 1, absence was coded 0. ^b^ Symptoms of depression were assessed using the Beck Depression Inventory-Second Edition [47]. ^c^ Childhood and adult problems with attention and hyperactivity were assessed using an 18-item self-report scale for the assessment of attention deficit hyperactivity disorder (ADHD) for adults [53]. ^d^ We screened for attention-deficit disorder using the short version of the Wender Utah Rating Scale [52]. ^e^ Impulsivity was assessed using the German translation of the Barratt Impulsiveness Scale (Barratt & Patton, 1995). ^f^ We screened for harmful alcohol consumption using the Alcohol Use Disorders Identification Test [46]. ^g^ Nicotine dependence was assessed using the Fagerström Test for Nicotine Dependence [45]. ^h^ Alexithymia was assessed using a German version of the Toronto Alexithymia Scale-26 [49]. ^i^ Use of adaptive and maladaptive emotion regulation strategies was assessed using the FEEL-E questionnaire [51]. ^j^ Emotion regulation strategies reappraisal and suppression were assessed using Emotion Regulation Questionnaire [50]. ^k^ Attachment style was assessed using the attachment-related anxiety and attachment-related avoidance subscales of the Experience in Close Relationships-Revised Scale [48]. ^y^ Higher values indicate greater problems.

## Supplementary Materials

The following are available online at http://www.mdpi.com/2077-0383/8/2/157/s1, Additional Analyses.
