# Supplementary material for: Same Same but Different: A Clinical Characterization of Men with Hypersexual Disorder in the Sex@Brain Study
Source: J Clin Med. 2019 Jan 30;8(2):157. doi: 10.3390/jcm8020157 (PMC6406591; doi:10.3390/jcm8020157)
Supplement: Supplementary file 1 [file jcm-08-00157-s001.pdf]

Supplementary

Additional Analyses

To control for the effect of comorbid disorders we replicated the analyses described after excluding all participants with a lifetime psychiatric history (HD = 21; HC = 22). Overall the pattern of results was similar to that for the whole sample. In contrast to the whole sample, no difference between groups could be seen in regard to child traumatization (CTQ/$t(40) = 0.91$, $p = .37$), sexual inhibition due to threat of performance failure (SIS1/$t(41) = 1.1$, $p = .28$), sexual inhibition due to thread of consequences (SIS2/$t(41) = 1.9$, $p = .07$) and consumption of child abusive images (Fisher's exact test ($N = 41$), $p = .49$). In contrast to the whole sample differences could be seen in relationship status (Fisher's exact test ($N = 31$), $p = .03$) more healthy controls were in a relationship than men with HD.
